# Harnessing *C. elegans* as a Biosensor: Integrating Microfluidics, Image Analysis, and Machine Learning for Environmental Sensing

**DOI:** 10.3390/s25216570

**Published:** 2025-10-25

**Authors:** Davin Lemmon, Gabriel Lopez, Jarrod Schiffbauer, Sebastian Sensale, Gongchen Sun

**Affiliations:** 1 Department of Biomedical Engineering and Chemical Engineering, Klesse College of Engineering and Integrated Design, University of Texas at San Antonio, San Antonio, TX 78249, USA; 2 Department of Physics, School of Science, Indiana University Indianapolis, Indianapolis, IN 46202, USA; 3 Department of Physical and Environmental Sciences, Colorado Mesa University, Grand Junction, CO 81501, USA

**Keywords:** *C. elegans*, biosensor, microfluidics, AI

## Abstract

Environmental contamination is becoming an increasingly evident risk to human health worldwide. The small, free-living nematode *Caenorhabditis elegans* (*C. elegans*) has become a compelling model organism for environmental toxicity studies in recent years, owing to its numerous advantages, including its transparent body, small size, well-characterized biology, genetic tractability, short lifespan, and ease of culture. Several assays have been developed using *C. elegans* to enable a better understanding of toxicant effects, from whole-animal to single-cell levels. While these methods can be extremely useful, they can be time-consuming and cumbersome to perform on a large scale. Recent advances in microfluidics have adapted many of these assays to enable high-throughput analysis of *C. elegans*, greatly reducing time and resource consumption while increasing efficiency and scalability. Further integration of these microfluidic platforms with machine learning expands their analytical capabilities and accuracy, revolutionizing what can be achieved with this model organism. This article will review the physiological basis of *C. elegans* as a model organism for environmental toxicity studies, and recent advances in integrating microfluidics and machine learning which could lead to using *C. elegans* as a promising living biosensor for environmental sensing.

## 1. Introduction

Contamination of natural resources is becoming an increasingly pertinent issue, as more people are experiencing its effects due to rapid industrialization and continued population growth [1]. Among the resources being affected, the persistent degradation of soil and water is increasingly threatening human health and causing significant environmental impacts [2,3]. Soil and water are critical necessities to maintaining a supply of food and sustaining a population and are not only necessary for humans but are required for the ecosystem as a whole to thrive and flourish [4].

Although 71% of the Earth’s surface is covered by water, freshwater makes up only about 2.5% of it [5,6,7]. Of this small amount, 98.8% is held in groundwater or frozen in glaciers and ice caps in the Arctic and Antarctic regions [5,7]. Thus, only 0.3% of all water on Earth is available for human consumption, and it is estimated that only 60% of monitored water bodies are considered to meet good ambient water quality standards [7,8]. Water pollution alone accounted for 1.4 million deaths in 2019, a problem that is exacerbated by the lack of reliable, representative water quality data [9,10]. Current water quality monitoring techniques primarily involve either in situ measurements or remote sensing [9]. These monitoring techniques can provide valuable data on water quality; however, each has limitations that prevent them from being as effective as needed. In situ measurements are labor-intensive, time-consuming, and costly, and fail to capture spatial and temporal variations in quality indices [3,11]. Remote sensing, on the other hand, can provide large-scale and frequent measurements but is limited by its requirement for specific weather conditions, atmospheric interference, and the inability to accurately determine parameters such as dissolved oxygen and biochemical oxygen demand [3,12].

Globally, there are over 10 million sites with contaminated soil, and over 50% of them are tainted with heavy metals and/or metalloids [13]. Additionally, an estimated 45 million hectares of agricultural land are affected by salinization, with this area increasing by 200,000 to 500,000 hectares annually [14]. The current methods used for evaluating soil quality involve laboratory-based techniques, real-time continuous soil monitoring (RTCSM), remote sensing, and visual assessment [2,14,15]. Each of these methods suffers from drawbacks in similar ways to those of water quality monitoring methods. For instance, laboratory-based techniques are time-consuming, labor intensive and costly; RTCSM uses sensors that require constant calibration and can be affected by the complex nature of soil; remote sensing techniques require bare soil to be most effective and can be influenced by multiple soil properties; and visual assessment is only able to provide qualitative and subjective data [2,13,14,15,16]. Microfluidic methods have also been developed for automated heavy metal ion detection [17].

Due to these limitations and the need for low-cost methods that are both effective and efficient, research efforts have increasingly turned to biological organisms for environmental sensing. These organisms, referred to as bioindicators or biomonitors, respond to alterations in their surroundings, offering a way to assess environmental quality through measurable biological changes [18,19]. Bioindicators reflect the biotic or abiotic state of the environment [18], while biomonitors provide quantitative data on pollutant levels, making them useful for both ecological assessments and pollution tracking [20]. Compared to electronic sensor networks, biological monitoring is often more cost-effective, particularly for large-scale or long-term studies, and certain organisms can detect pollutants at concentrations below the detection limits of conventional analytical instruments [18].

Microbial assemblages, plants, and various animal species all play roles in environmental sensing, with microbial communities serving as rapid responders due to their short generation times, plants such as lichens serving as air quality monitors, and animals like honeybees, bivalves, and earthworms accumulating contaminants that indicate pollution levels [18,21,22]. These organisms can be incorporated into biosensors, which integrate a bioreceptor with a transducer to recognize a target molecule and convert the recognition event into a measurable signal [23,24]. The ability of biosensors to integrate environmental exposure over time makes them particularly valuable for assessing chronic, low-level pollution, which might otherwise go undetected [25]. For example, whole-cell biosensors using various bacterial cells can be developed into imaging-based sensors for medium-throughput detection of numerous toxicants, including heavy metals, pesticides, and other organic pollutants [23,26,27,28,29].

Single organisms, such as microbial cells, nematodes, and honeybees, provide direct physiological and behavioral responses to environmental stressors, making them highly sensitive early warning indicators [30]. An example of single-organism sensors are microbial electrochemical sensors, which meet the high-throughput requirements for soil and water monitoring by using electrical signals altered by chemical reactions between an analyte and electroactive microorganisms and/or biofilms to sense the presence of contaminants [31,32,33,34]. These sensors have been effectively used for a variety of purposes, including toxicity, pathogen, and corrosion monitoring in water, as well as heavy metal and pesticide detection in soil [29,34]. They are typically low-cost, easy to operate, and highly sensitive. However, they lack selectivity, as multiple compounds may trigger the same electrical signal change, potentially leading to false positives in the sensing readout [35]. To address the need for multiplexed detection in biosensors that allows for different environmental agents to be distinguished, multicellular organisms have emerged as potential solutions. Their more sophisticated physiological structure may enable them to react differently to various stimuli they encounter.

The multicellular free-living soil nematode *Caenorhabditis elegans* (*C. elegans*) is a promising organism for use as a biosensor. Due to its genetic similarities to humans and its utility for studying complex biological processes, *C. elegans* is a widely used model organism in biological research [36]. Physiological complexity enables *C. elegans* to function as a sophisticated biosensor. It possesses metabolically active digestive, reproductive, endocrine, sensory, and neuromuscular systems, allowing it to respond to contaminants as a functional multicellular organism [37].

This review article aims to argue that *C. elegans* could be an attractive candidate for this purpose. Recent research has shown that *C. elegans* is an effective model for evaluating the toxicity of environmental contaminants in both soil and water ecosystems [38]. In the following sections, we will review (1) the physiological basis of *C. elegans* as a biosensor, (2) its integration with high-throughput microfluidic handling tools, and (3) artificial intelligence (AI)-driven image analysis techniques to deep phenotype *C. elegans* for environmental sensing. In the final section, we will discuss two recent examples that highlight the integration between living organism *C. elegans*, advanced microfluidic tools, and machine learning (ML)-enhanced image analysis for environmental contamination studies. Overall, we aim to demonstrate the feasibility of an integrated system between living organism *C. elegans*, advanced microfluidic tools, and machine learning-enhanced image analysis as a promising sensitive and multiplexed platform for environmental sensing.

## 2. *C. elegans* as Biosensors

*C. elegans* offers several experimental advantages that make it particularly suitable for environmental toxicity studies. Practical advantages include its small size, transparency, ease of culture, rapid growth, large brood size, and well-mapped cell lineage [39]. As a non-parasitic worm, large populations can be grown in minimal space, enabling large-scale testing at significantly lower financial and ethical costs than traditional models [40]. Its transparent body permits direct visualization of organs, contaminant internalization, and cellular markers, making it possible to track the fate of ingested compounds and observe fluorescently tagged transgenic proteins using microscopy [41]. Most importantly for toxicity assessment, *C. elegans* has a short life cycle of only 3.5 days [42], enabling observation of contaminant effects on lifespan, reproduction, and transgenerational effects in relatively short timeframes. Due to prolific hermaphroditic reproduction and low-cost maintenance, large populations with identical genetic backgrounds can be propagated quickly and efficiently under laboratory conditions [43]. The flexibility of culturing *C. elegans* on solid media or in liquid medium using various formats (Petri dishes, tubes, or well plates) allows for contaminant exposure through different routes (injection, feeding, or soaking), enabling both acute and chronic toxicity studies [38].

Sensitivity and predictive value further enhance its utility as an environmental biosensor. *C. elegans* is more sensitive to metals and pesticides than other recommended Environmental Risk Assessment (ERA) models and responds to most nanomaterials, making it feasible for early monitoring of environmental compounds [38,43]. The fully sequenced genome and large library of available transgenic strains facilitate genetic studies [44]. Around 40% of the 20,000 genes present in *C. elegans* are homologous in humans, and most of the protein domains present in humans can be found in the worms as well [45,46]. Due to this homology, various integral metabolic pathways are distinctly similar between worms and mammals, allowing for the metabolic effects of compounds to be incorporated into toxicological studies [47]. Comparative studies of lethal concentration (LC50) values for *C. elegans* of both heavy metals [48] and organophosphates [49] have shown paralleled ranking for rat median lethal dose (LD50) values. These findings indicate that morbidity and mortality measurements coupled with morphology analyses in *C. elegans* may have the potential to predict mammalian toxic responses. Also, while the nervous system of *C. elegans* is vastly simpler than that of humans, it shares fundamental principles of neural development and function, offering crucial insights into conserved processes found in humans [50,51].

Numerous assays have been developed to study toxicity mechanisms and evaluate health risks of environmental contaminants using *C. elegans* [41,52]. These methods can be categorized into two broad groups: those that focus on whole-organism endpoints and those that focus on in vivo biological markers. Whole-organism endpoints are outcomes that can be observed in an entire organism to indicate a disease state. These focus on assessments of lethality, growth rate, reproduction, and locomotion [41]. The assessment of lethality serves as a basic measure of toxicity and involves observing whether the nematode dies when exposed to the contaminant [52]. The effects of toxicants on the growth of *C. elegans* can be assessed by comparing worm development before and after exposure to control groups, typically done through microscopic imaging of body size [41]. Certain high-throughput equipment, such as the Complex Object Parametric Analyzer and Sorter (COPAS), can also measure optical density as an endpoint indicator of growth [52,53]. To evaluate reproductive effects, brood size is assessed after contaminant exposure [52]. Changes in movement and behavior of *C. elegans* after exposure can be determined by single worm imaging and computer tracking methods. This assay can indicate neurotoxicity or general stress [54,55]. Additional whole-organism endpoints include chemotaxis assays to determine whether volatile compounds are attractive or aversive to the worms [56], and feeding behavior assessments to determine if toxicants inhibit feeding [57].

In vivo molecular markers provide deeper understanding of the physiological state and how it is affected by toxicant exposure [41]. Gene expression analysis quantifies transcriptional changes in contaminant-exposed nematodes [41,57,58]. Multiple methods can assess gene expression, including RNA sequencing, real-time qPCR, and live fluorescent reporter assays (e.g., GFP) [41]. Protein expression changes can be evaluated using ELISA and Western blots [59]. Oxidative stress can be determined by measuring antioxidant enzyme activity through quantification of reactive oxygen species, using fluorogenic dyes or transgenic strains with live fluorescent reporters [41,57]. Metabolomics and metabolic profiling determine toxicant mechanisms by analyzing metabolites using liquid chromatography mass spectrometry (LC-MS/MS) or gas chromatography mass spectrometry (GC-MS) to identify statistically significant differences between control and exposed groups [60,61,62]. DNA damage can be assessed using qPCR, comet assays, TUNEL staining, and transgenic strains, providing understanding of genotoxic effects [63,64,65]. Mitochondrial toxicity can be evaluated through measurements of mitochondrial morphology, membrane potential, ATP levels, and respiratory chain enzyme activity, which are critical indicators of cellular energy disruptions [66,67,68,69].

**Figure 1:**
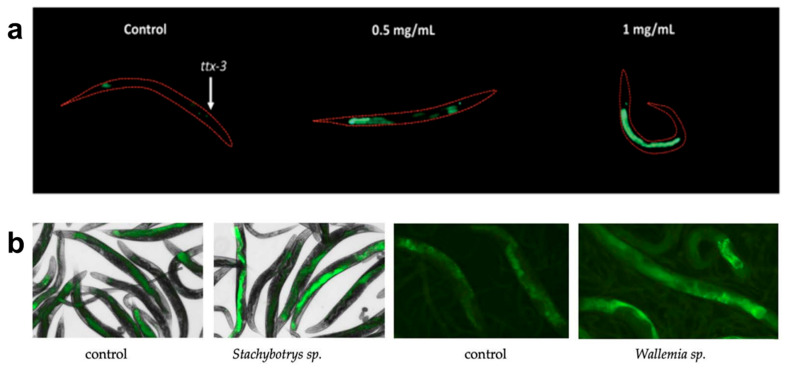
Examples of *C. elegans*’ use as sensor. (**a**) Representative images of the *C. elegans* strain (*agIs219*) fluorescing when exposed to different concentrations of perchlorate for 24 h, adapted from [74]. (**b**) Effects on the fluorescent responses of the *cyp-34A9*::GFP strain to airborne exposure to fungal cultures of *Stachybotrys* sp., and of the *sod-4*::GFP strain exposed to the fungal cultures of *Wallemia* sp., adapted from [56].

Using these methods, *C. elegans* can effectively detect and study the effects of various hazardous compounds, including heavy metals, pesticides, nanomaterials, hydrophilic contaminants, biological toxins, and chemical surfactants. Heavy metals like hexavalent chromium have been detected using transcriptional response assays, which analyze changes in gene expression in stress-response pathways such as metallothioneins and heat shock proteins [57]. Pesticide exposure has been evaluated using locomotion and reproductive assays, where neurotoxic effects are measured through changes in movement patterns and brood size [70,71]. Nanomaterial toxicity has been studied by measuring reactive oxygen species levels, mitochondrial dysfunction, and fertility impairments, all of which indicate nanomaterial-induced cellular stress [43,62,72]. An example of nanomaterial effects on fertility is reported by Ma et al., where TiO2 material effects are observed in the vulval integrity of aging worms [73]. Hydrophilic contaminants, such as perchlorate, have been detected using GFP reporter gene assays targeting oxidative stress and metabolic disruption pathways, providing real-time visualization of cellular responses to toxicant exposure [74]. Figure 1a shows how the transgenic strain *agIs219* fluoresces when exposed to different concentrations of perchlorate for 24 h. Organic pollutants have been investigated using behavioral assays and metabolic profiling, where locomotion assays reveal disruptions in neuromuscular function and shifts in metabolomic signatures indicate biochemical changes [61,75]. Furthermore, *C. elegans* has been used to monitor indoor air pollutants such as fungal toxins, as alterations in feeding behavior, oxidative stress marker expression, and neurodegenerative-like symptoms have been identified after exposure [56]. The response of two transgenic strains expressing GFP in response to oxidative stress can be seen in Figure 1b, where different strains undergo airborne exposure to fungal cultures and experience oxidative stress.

## 3. High-Throughput Analysis Using Microfluidics

**Figure 2:**
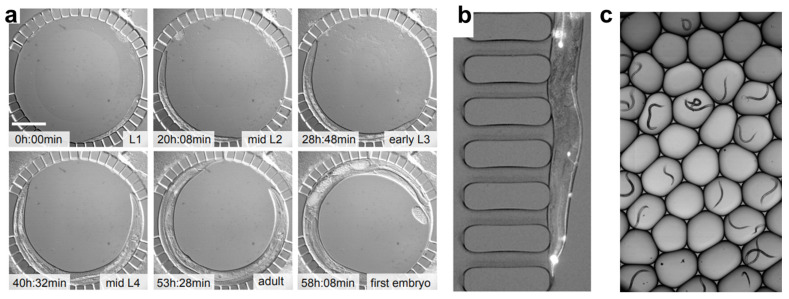
Examples of the use of microfluidics in *C. elegans* analysis. (**a**) A microfluidic chamber that enables long-term studies of larval development of *C. elegans*, adapted from [82]. (**b**) Worms are encapsulated in droplets for continuous-flow fluorescence expression analysis, adapted from [98]. (**c**) Using multiple suctions channels a single worm is captured for fluorescence imaging of touch neurons and their processes, adapted from [100].

While *C. elegans* can serve as an effective sensor for detecting environmental contaminants, many of the assays described in the previous section are time-consuming and low-throughput. These traditional manual methods are limited by inefficiencies due to labor-intensive sample preparation, limited reproducibility, and the need for significant user oversight [76]. Therefore, there is a need to streamline these processes and provide high-throughput, parallel sample analysis. Due to the small size of the worms, microfluidics provides an effective platform for high-throughput worm handling and analysis, allowing for precise control of experimental conditions, automation of assays, and real-time data acquisition [77]. Microfluidics enables the simultaneous processing of multiple samples with minimal resource consumption, significantly improving both the efficiency and scalability of analysis platforms [78,79]. Additionally, it allows for the integration of multiple detection modalities, such as optical, electrophysiological, and mechanical, thus enhancing the accuracy and depth of toxicological assessments [77,80]. The ability to analyze and manipulate *C. elegans* in a controlled microenvironment also enables the study of long-term toxicant exposure, behavioral phenotyping, and cellular responses [76,81]. Keil et al. developed a device (Figure 2a) that allows for the observation of up to ten *C. elegans* larvae as they develop from hatching to adulthood, enabling the study of cellular behavior during larval development [82]. Aside from their customizability and high-throughput capabilities, microfluidic methods are cost-effective, disposable, and user-friendly, requiring minimal training for operators compared to traditional analytical methods [78]. The continued advancement of microfluidic technologies offers the potential to transform environmental toxicology by providing more reliable, reproducible, and high-throughput screening methods for the model organism *C. elegans*. In this section, we review various microfluidic technologies that enable high-throughput analysis of *C. elegans* with specific focus on worm-environment interactions, which could be the foundation for using high-throughput *C. elegans* assays as a reliable environmental sensing solution.

### 3.1. Behavioral Assays

Microfluidic platforms provide precisely controlled environments for studying *C. elegans* behavior, enabling high-resolution monitoring of locomotion, chemotaxis, and feeding responses [83]. Unlike traditional Petri dish or multi-well plate assays, microfluidic devices allow for tight spatiotemporal control of chemical gradients and real-time behavioral tracking using integrated imaging systems [84]. A microfluidic device designed by Kopito and Levine called ‘WormSpa’ enables spatiotemporal surveillance of *C. elegans* responses to environmental cues through precise control of factors like temperature, chemical gradients, and food availability [85]. This allows for continuous observation of the worms over long periods without inducing stress or compromising normal physiological functions. Other well-controlled fluidic systems can create varying flow speeds and viscosities to investigate the effects of toxicants on worm mobility, with some having channels that mimic terrain-like constraints to provide more physiologically relevant evaluation of locomotor behavior [86]. Lockery et al. designed the “artificial dirt” chip, which consists of an array of pillars at different spacings to better approximate the complexity of worm locomotion in natural substrates [87]. These assays can be further refined with integrated high-speed imaging systems and automated tracking software, which provide quantitative measurements of velocity, body bends, and waveform patterns [88]. Shi et al. developed a device to enable the evaluation of movement and fluorescence imaging analysis of individual *C. elegans* continuously and automatically in a high throughput manner at single animal resolution [89]. This allowed for the study of how neurotoxins, like 6-hydroxydopamine (6-OHDA) and 1-methyl-4-phenylpyridinium (MPP^+^), induce mobility defects that correlate with selective degeneration of dopaminergic neurons and increased oxidative stress in a dose dependent manner. Computer vision (CV) and ML algorithms can further automate behavioral analysis, reducing variability and increasing throughput (see Section 4).

Whole-organism responses to environmental contaminants can be studied through worm chemotaxis assays. Microfluidic platforms greatly improve chemotaxis experiments by creating stable, reproducible chemical gradients, allowing precise control of the spatial and temporal distribution of attractants or repellents. Albrecht et al. developed a fluidic controlled micropillar array device, demonstrating the behavioral response dynamics to spatiotemporally controlled release of attractive odor (isoamyl alcohol) in *C. elegans* at the individual-worm level [90]. Unlike traditional agar-based setups, microfluidic devices allow for dynamic gradient adjustments, providing real-time assessments of how worms handle contaminant-laden environments. Song et al. demonstrated how their microfluidic device allows two different chemical solutions to be introduced to individual chambers with short transition times [81]. These systems also permit single-worm tracking, reducing variability and enhancing statistical power.

Microfluidic methods have also enhanced characterization of subtle worm behaviors. Using microfabricated chambers with controlled bacteria suspensions, pharyngeal pumping rates and feeding behaviors of individual worms can be determined with high precision [91]. Devices like ‘WormSpa’ and ‘C.L.I.P’ (Continuous Live Imaging Platform) enable long-term imaging of these physiological activities and provide insights into metabolic and neurological impairments by enabling real-time monitoring of feeding inhibition caused by toxicants [85,92]. Furthermore, integrating optical and electrical sensing techniques into microfluidic platforms enhances the accuracy of detecting subtle changes in physiological activity, which would be difficult to discern using conventional approaches [93,94,95].

### 3.2. High-Resolution Imaging

Microfluidic systems can integrate advanced imaging techniques, including confocal microscopy, single-molecule fluorescence in situ hybridization (smFISH), and high-speed live imaging, providing high-resolution visualization of *C. elegans* at the cellular and subcellular levels [96,97,98]. The ability to precisely position and immobilize worms without anesthetics is a key advantage of microfluidic imaging, as it avoids interference with biological processes [99]. Microfluidic devices achieve this using gentle suction or structured channels to hold worms in place, ensuring stable and repeatable imaging conditions while preserving physiological states. Rohde et al. used suction channels in their device design (Figure 2b) to gently immobilize worms for fluorescence imaging of touch neurons and their processes [100].

Various features within microfluidic devices significantly improve efficiency in toxicity studies by allowing parallel imaging of multiple worms. For example, a microfluidic droplet device developed by Yan et al. (Figure 2c) allows high-throughput encapsulation of single worms and fluorescence imaging at a rate of 0.5 s per worm, while a parallel multichannel microfluidic device developed by Hulme et al. foregoes high throughput in favor of long-term imaging capabilities for a moderate number of worms [98,101]. These systems enable tracking of dynamic cellular events such as apoptosis, mitochondrial activity, and neuronal degradation over extended periods [82,102]. Due to their low residence times, microfluidic systems also provide the ability to rapidly switch exposure conditions of chemical stimuli, enhancing the study of acute and chronic toxicant effects and providing insights into adaptive cellular responses and stress mechanisms [88]. Additionally, microfluidic-enabled high-resolution imaging allows real-time monitoring of neurotoxic effects, metabolic dysfunction, and tissue damage, reducing the need for endpoint staining and improving the accuracy of phenotypic assessments [103,104].

### 3.3. Drug Screening and Toxicology Studies

Microfluidic platforms provide the capability of automating dosing systems in order to precisely regulate the exposure duration and concentration of toxicants or drugs [83]. This enables accurate and reproducible exposure of *C. elegans* to toxicants and drugs for detailed dose–response profiling, which provides a level of control that is essential for identifying toxicity thresholds and assessing drug efficacy [85]. To assess the uptake and toxicity of silver nanoparticles (AgNPs), Kim et al. devised a device to precisely control the concentration of AgNPs (0–1 mg/L) worms were exposed to and allow for the evaluation parameters such as length, moving distance, and fluorescence from a specific reporter gene [105]. The phenotype characterized in the presence of AgNPs, a shorter body, longer moving distance and highly expressed reporter gene, was different from that of gold nanoparticles and heavy-metal ions, indicating that this device could potentially serve as a rapid and specific nanoparticle detection or nanotoxicity assessment system. Using a microfluidic chip, Letizia et al. developed a method of predicting the effect of doxycycline on *C. elegans* development [106]. With the chip’s ability to precisely control the environment around the worms, they identified a relationship between temperature, food concentration, and doxycycline dose on worm development, allowing for a better understanding of the effects of the drug [106]. Salam et al. used a microfluidic device with an integrated electric field to study the effects of neurotoxins, 6-OHDA, 1-methyl 4-phenyl 1,2,3,6-tetrahydropyridine (MPTP), and rotenone on the electrotactic swimming behavior of the worms [107]. Animals exposed to these toxins were found to experience reduced speed with intermittent pauses, abnormal turning and slower body bends, a phenotype that was also observed in mutants affecting sensory and dopaminergic neurons. This characterization demonstrates that the electrotactic swimming response of *C. elegans* can be highly sensitive and reliable in detecting neuronal abnormalities.

Additionally, microfluidics permits combinatorial testing, where multiple drugs or toxicants can be introduced simultaneously or sequentially to assess synergistic or antagonistic interactions [100]. A microfluidic device and drug testing method produced by Ding et al. provides the ability to discover effective drug combinations of available anthelmintics on *C. elegans* [108]. Having the ability to generate chemical gradients within the chambers of microfluidic devices allows for the simulation of complex environmental exposures that more accurately reflect real-world conditions [76]. They also allow for continuous monitoring of drug absorption, metabolism and excretion at the single-worm level, factors that are important for studying pharmacokinetics [109]. This capability offers invaluable information to assess the biotransformation of xenobiotics and their long-term impacts on organismal health.

### 3.4. Metabolic Studies

Microfluidic systems enable precise delivery and manipulation of chemicals in the environment surrounding worms. This allows more accurate control of toxicant concentration and exposure duration compared to conventional assays, as diffusion and evaporation from well-plates or Petri dishes often lead to inconsistencies [84]. With the level of control and precision that microfluidics provides, dose-dependent metabolic responses to various contaminants and drugs can be better understood [104]. Studying these responses offers valuable insights for developing treatment strategies for humans exposed to the same toxicants due to the worm’s genetic homology to humans [110].

The efficacy of drugs targeting metabolic disruptions caused by contaminants can be effectively evaluated through microfluidic metabolic analysis. Wen et al. constructed a microfluidic device to enable long-term culture and flexible manipulation of *C. elegans* [111]. Using this device, they studied the lifespan-extending activity of polydatin on worms treated with copper ion to induce oxidative stress. After being exposed to copper ion, the lifespan, mobility behaviors and the expression of inducible oxidative stress protein were characterized. An increase in oxidative stress coupled with a decrease in the lifespan of the exposed worms suggested that an oxidative stress pathway in the aging process may have been activated. This, along with the extension in lifespan of the worms treated with polydatin implies that the polydatin provides a protective response to oxidative damage in the worms. The impact of drugs on restoring normal metabolic function can be assessed by precisely controlling contaminant exposure and administering potential therapeutic compounds on-chip [112]. Stress responses can also be used in concert with microfluidic devices to study the dose-dependent metabolic impact of contaminants and the potential protective effects of other compounds by monitoring indicators such as oxidative stress-sensitive GFP expression [113].

Additionally, quantitative proteome analysis of single *C. elegans* is enabled through digital microfluidics (DMF) proteomics [114]. This technology combines single-pot solid-phase enhanced sample preparation (SP3) with high-field asymmetric-waveform ion mobility spectrometry (FAIMS) and mass spectrometry to identify thousands of proteins from a single worm. This data can then be used to study the effects of contaminants on protein abundance, which directly reflects metabolic changes in the organism [114].

### 3.5. Long-Term On-Chip Culture

Microfluidic devices enable stable and controlled conditions for *C. elegans* culturing over days to weeks, enabling long-term studies of development, aging, behavior, and responses to various stimuli [86]. These systems offer the potential to study environmental toxicant effects during the entire lifespan of an organism. The devices provide continuous flow of liquid culture media through microchannels and chambers housing the worms, ensuring constant nutrient supply while removing metabolic waste products [115]. Microfabricated chambers can house individual worms or embryos, preventing overcrowding and resource competition, which allows for long-term studies of single worms throughout their lifespan. Pan et al. developed a microfluidic device to isolate individual worm embryos and allow long-term live imaging [116]. This enabled the study of embryo responses to mechanical and chemical stimulation and how those stimuli affect worm development.

Filters or narrow channels can be fabricated at microchamber outlets to retain adult worms while allowing smaller progeny, such as eggs and larvae, to be flushed out by continuous flow. This feature is well visualized in the ‘WormSpa’, where flow through the chamber housing the worm not only replaces nutrients and removes waste, but also drives eggs into a separate chamber area [85]. Many microfluidic devices are fabricated using polydimethylsiloxane (PDMS), a biocompatible and gas-permeable polymer that allows passive exchange of essential gases [117]. This assists in maintaining suitable physiological environments over long periods. Furthermore, microfluidic devices can be integrated with temperature control systems to maintain stable and optimal temperatures for development and survival throughout long-term studies, ensuring consistent development rates while minimizing non-toxicant related stress [115].

## 4. AI-Driven Image-Based Sensing for Deep Phenotyping of *C. elegans*

Microfluidic platforms enable high-throughput collection of *C. elegans* phenotypic data, often presented as high-content imaging datasets. However, analyzing these images to study *C. elegans* physiology still presents significant challenges to date [76]. Manual processing using semi-automated, open-source platforms such as ImageJ [118] remains a primary method for phenotyping; yet, this approach is labor-intensive, time-consuming, and prone to subjectivity [119]. Over the past decade, AI has emerged as a powerful tool to overcome these limitations [120]. AI-driven image analysis represents a major advancement across multiple fields, from scientific research to agriculture and medicine [121]. In *C. elegans* studies, it has significantly improved the efficiency and accuracy of phenotypic analysis, enabling faster and more precise classification than traditional manual methods and facilitating the identification of subtle phenotypical changes caused by environmental toxicants [122,123].

**Figure 3:**
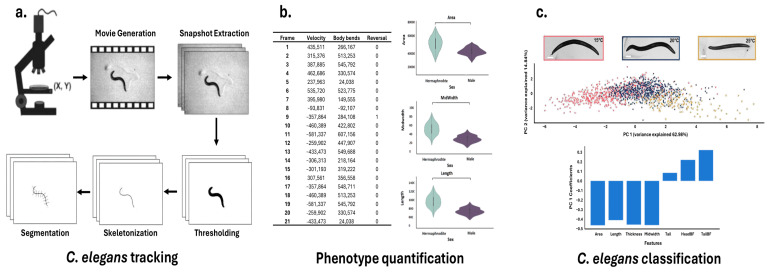
Typical workflow for *C. elegans* Tracking, Phenotype Quantification, and Classification. A comprehensive pipeline for analyzing *Caenorhabditis elegans* behavior and morphology. (**a**) *C. elegans* tracking workflow, including movie generation, snapshot extraction, thresholding, skeletonization, and segmentation. (**b**) Quantification of phenotypic features such as velocity, body bends, and reversals across video frames, along with morphological metrics (area, midwidth, and length) stratified by sex. (**c**) Classification of *C. elegans* based on extracted features using principal component analysis (PCA), with representative images showing temperature-dependent phenotypes (15 °C, 20 °C, 25 °C) and feature contribution bar plots. Adapted from [128,129].

The massive and heterogeneous datasets generated by microfluidic platforms pose significant analytical challenges that exceed the capabilities of traditional methods [124]. Optical signals are the most prevalent data type, and lend themselves naturally to CV [125]. Deep Learning (DL), specifically convolutional neural networks (CNNs) and generative adversarial networks (GANs), dominates the field of computer vision, enabling rapid, consistent analysis of vast visual datasets in *C. elegans* phenotyping studies [120]. These techniques have demonstrated remarkable utility in three key areas: accurate motion tracking and quantification to study behavioral patterns [126], automated image and video analysis for phenotypic quantification and growth measurements [127], and pattern recognition in large datasets to generate new hypotheses about biological mechanisms [120] (Figure 3).

### 4.1. Tracking C. elegans Motion Using AI

Traditional *C. elegans* tracking methods rely on extracting individual frames from video recordings and applying thresholding techniques to differentiate the worms from their background [129]. These methods often involve open-source image processing tools such as ImageJ 1.54p [118]. Advances in Machine Learning (ML) and Computer Vision (CV) led to the development of tracking software with enhanced automation capabilities such as Tracker 3.0 [129,130]. This software enabled the user to track the motion of one worm at a time; however, the thresholding technique it uses fails when occlusion occurs, causing it to lose track of the worm in multi-worm environments [129,131,132].

The need to manage and analyze multiple worms prompted the development of the Multi-Worm Tracker (MWT) software [133]. This tool integrates a real-time image analysis software, based on segmentation tracking with bounding box constraints, with an offline post-processing software to enable comprehensive analysis of multiple worms within a single Petri dish [133]. Unlike other high-throughput behavioral screening methods that parallelize multiple well plates with limited image-processing capabilities [133], MWT excels in tracking a broad range of individual behavioral patterns, offering greater versatility for high-throughput analysis [134]. However, this software proved inadequate when confronted with challenging scenarios such as complex postural configurations involving significant self-overlap (coiling) [133].

Roussel et al. developed a deformable model-based tracking algorithm to improve accuracy in cases of extreme self-overlap and complex posture. Implemented within the WormLab software (MBF Bioscience, Williston, VT, USA), their methodology implements energy minimization algorithms to iteratively align a flexible shape model to the worm’s body across frames, enabling robust, continuous tracking of multiple worms even under complex postural configurations or partial occlusion [132]. While this approach performs well in controlled environments with high contrast between the worms and the background, its performance may degrade under typical laboratory conditions where noise, variable illumination, and cluttered backgrounds introduce additional segmentation challenges [135]. To deal with this problem, Javer et al. developed the Tierpsy Multi-Worm (TMW) 2.0 software. This software employs adaptive thresholding to differentiate worms from their background based on variations in light intensity [136]. Subsequently, feature extraction is performed to measure the shape of the worm from the background through another thresholding step. This operation classifies pixels as either worm or background based on whether their intensity exceeds a defined threshold value, generating a binary (black and white) image [137].

Recent DL methodologies incorporate pretrained models in conjunction with transfer learning strategies, enabling the repurposing of high-level feature representations acquired from large-scale datasets [138]. This approach facilitates efficient adaptation to novel, domain-specific tasks by significantly reducing the need for extensive labeled data and computational resources, while maintaining high performance in specialized applications. An example of such software is Deep-Worm-Tracker (DWT) 1.0, an end-to-end DL model that combines the You Only Look Once (YOLOv5) object detection model [139] and the Strong Simple Online Real Time Tracking (Strong SORT) backbone for accurate, real-time tracking and feature extraction [139,140]. After bounding-box tracking, segmentation and skeletonization algorithms are applied to the predicted bounded regions for each worm ID, avoiding the capture of unnecessary background noise [135] (Figure 3a). One-shot architectures like YOLO perform object detection in a single step, using predefined anchor boxes on an image grid to predict object presence. In contrast, two-shot detectors employ a two-stage approach: first using a region proposal network (RPN) to identify potential regions of interest, then using a second network to refine these proposals into final predictions [141]. While one-shot methods, such as DWT, generally require less computational power than two-shot approaches (TMW), two-shot methods, such as WormSwin 1.0, typically achieve greater precision, particularly in complex scenes [141]. This advantage has made two-shot networks like Mask R-CNN particularly popular for instance segmentation tasks [142].

### 4.2. Quantifying C. elegans Phenotypes Using AI

To transform qualitative observations into robust quantitative data, acquired images undergo feature extraction, often through house-made codes [143]. During the process known as ‘segmentation’, the worm’s body is reduced to a one-pixel-thick skeleton line, which is then subdivided into segments [144]. These segments enable the computation of parameters such as the center of mass (centroid) of either the entire organism or individual body sections, the body curvature, associated with the angles between adjacent segments [145], and the worm’s speed, by measuring the rate of change in the location of the worm’s centroid or points along its skeleton over time across sequential video frames [146] (Figure 3b). To ensure consistency and reproducibility in the extraction of these features, Hakim et al. introduced the software platform ‘WorMachine’ [128]. This tool automates the extraction of key phenotypic parameters from raw worm images, reducing user-dependent variability and enhancing the accuracy of quantitative measurements. Beyond basic morphological and locomotion analysis, WorMachine characterizes complex features like transparency and fluorescence-based metrics by analyzing fluorescence intensity and distribution within the worm’s body, processing overlapping bright-field and fluorescence images to quantify signals for assessing gene expression patterns and protein localization. Several other tools have also been developed to further streamline phenotype analysis, expanding the range of measurable traits and improving the accuracy of high-throughput studies [120,147,148,149]. Furthermore, recent technical advances have expanded phenotypic quantification to incorporate subcellular structures [126,150] as well as three-dimensional analyses [151].

### 4.3. Classifying C. elegans by Phenotype Using AI

Morphological phenotypes such as surface area, volume, length, maximum width, and fluorescent signals of reporter molecules allow for differentiation between wild-type and mutant worms, reveal deviations from expected additive effects in double mutants, and provide insights into the genetic interactions that shape worm morphology [150,152]. However, the extensive data generated from such analyses results in high dimensionality, complicating interpretation and downstream analysis [153]. To address the challenges of high-dimensional data, multiple techniques are commonly used, such as Principal Component Analysis (PCA) [154], t-Distributed Stochastic Neighbor Embedding (t-SNE) [155], Linear discriminant analysis (LDA) [156], Independent component analysis (ICA) [157], Non-negative matrix factorization (NMF) [158], and Uniform Manifold Approximation and Projection (UMAP) [159]. These methods reduce data complexity by projecting it into a lower-dimensional space, making analysis more tractable while preserving as much relevant information as possible [120,128]. For example, in toxin quantification studies, *C. elegans* are exposed to water samples with known toxin concentrations. Phenotypic data from multiple worms are collected, and Principal Component Analysis (PCA) is used to capture key variation in their responses (Figure 3c). Regression models are then employed to relate these principal component (PC) scores to toxin levels [76,160]. A typical model might take the form: Toxin Level = a * PC1 + b, where a and b are parameters fitted to calibration data from known standards.

In complex scenarios—such as time-series analysis, non-canonical pattern recognition [161,162] or image-based phenotyping- ML and DL are increasingly employed [163]. These approaches support tasks like phenotype classification [164], regression modeling [165], phenotype quantification [166], and anomaly detection [167,168]. Common ML implementations include Support Vector Machines (SVM), Random Forest (RF), and decision trees. Thomas et al. employed SVM to differentiate and classify videos of *C. elegans* exposed to a range of environmental conditions, utilizing the ksvm function from the kernlab package in R for this analysis [169]. Similarly, Martineau et al. applied SVM to predict lifespan and quantify health metrics, using the fitrsvm and predict functions in MATLAB R2025a [170]. Ribeiro et al. applied RF identify lifespan-extending chemical compounds for *C. elegans*, implementing this approach in Python 3.11.6 with the imblearn, numpy, and sklearn libraries, where the BalancedRandomForestClassifier function facilitated effective handling of class imbalance and large datasets [171]. Guo et al. developed 600 decision trees in Python to enhanced mutagenesis probability mapping, enabling efficient identification of causal mutations in *C. elegans* [172]. While these ML techniques excel in structured data analysis and classification, DL approaches offer distinct advantages for processing complex, unstructured data, further expanding the scope of *C. elegans* research.

**Figure 4:**
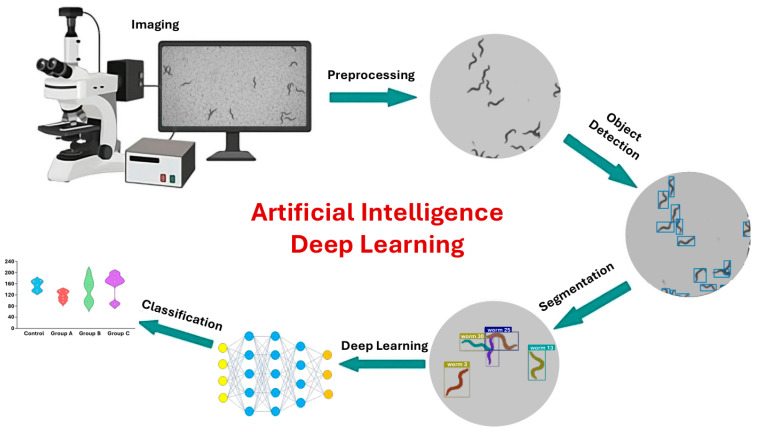
DL image analysis workflow. The process begins with image acquisition via microscopy, followed by preprocessing to enhance structural features. DL models are then applied for object detection and precise segmentation of regions of interest. Finally, the extracted features are classified to identify patterns and groupings within the data. This workflow demonstrates how AI can automate complex image analysis tasks and improve diagnostic accuracy in biomedical research (Adapted from [141,177,178]).

DL architectures such as CNNs or recurrent neural networks (RNNs) [126,173] can be applied directly to raw or minimally processed data. Self-supervised DL pipelines in computer vision and natural language processing enable precise prediction and quantification of features within images [174,175]. DL models can operate directly on pixel values, allowing them to capture complex worm behaviors, such as coiling, that are often missed by traditional key-point tracking or skeletonization methods [176]. The integration of AI into *C. elegans* phenotyping pipelines (Figure 4) represents a transformative leap, enabling precise, high-throughput, and nuanced analyses of behavior in response to environmental stimuli.

## 5. Integrating *C. elegans* Biosensor, High-Throughput Microfluidic Platform, and AI-Driven Image Analysis for Environmental Contaminant Analysis

As discussed in previous sections, *C. elegans* possesses several attributes that make it ideal for high-throughput phenotypic screening (HTS) using microfluidics: its compact size, transparent anatomy, low cost, ease of maintenance, and rapid life cycle [169]. These characteristics enable automated phenotypic analysis, where nematode responses to natural compounds and extracts can be systematically evaluated [146]. Measurable phenotypes include growth, lifespan, reproduction, locomotion, intestinal permeability, and microfluidics-specific parameters such as mechanical and electrical properties [179]. This diversity of phenotypic readouts generates rich datasets suitable for AI-driven analysis [124], which can predict correlations between physical characteristics and specific physiological or pathological states [180], establishing *C. elegans* as a powerful tool for environmental biosensing [120].

The optical transparency of *C. elegans* further enhances its utility by enabling multimodal imaging approaches. Fluorescent molecular reporters can be combined with behavioral imaging [181], producing integrated datasets from which AI models can learn correlations between genetic network activity and phenotypic behavior [128,182]. Machine learning and deep learning algorithms excel at extracting and integrating information from such multimodal datasets, enabling more comprehensive characterization of biological responses to environmental stimuli.

AI is also increasingly applied to microfluidic device design and operation itself. ML and DL models can predict performance parameters such as droplet generation efficiency or flow stability [183] optimize channel geometries and flow rates for specific assays [184], and enable intelligent control systems [124]. However, these engineering applications fall outside the scope of this review.

In the following subsections, we examine two representative examples that integrate *C. elegans* biosensors, high-throughput microfluidic handling, and AI-driven phenotyping for environmental contaminant analysis. These case studies illustrate the potential of integrated organism-device-AI platforms to achieve real-time, cost-effective, sensitive, and multiplexed environmental contaminant detection and health risk assessment.

### 5.1. High-Throughput Developmental Toxicity Screening with Automated Image Analysis

**Figure 5:**
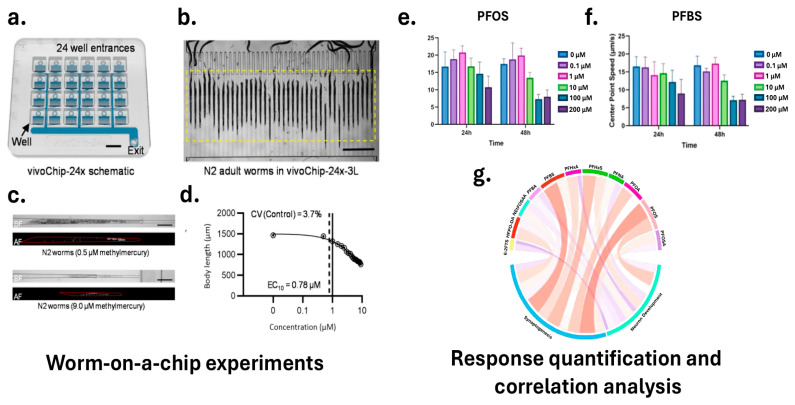
Integrated microfluidic and AI-based system for high-throughput screening in *C. elegans*. (**a**) The vivoChip-24x enables parallel immobilization and imaging of up to 1000 worms across 24 populations. (**b**) Brightfield image of adult worms aligned in the chip’s microchannels. (**c**) Representative brightfield and autofluorescence images show dose-dependent effects of methylmercury exposure. (**d**) Morphometric features such as body length, area, and volume were quantified using the vivoBodySeg model, with EC_10_ and LOAEL values identified. (**e**,**f**) Behavioral assays reveal significant changes in center point speed following exposure to PFOA and PFBS. (**g**) A chord diagram illustrating correlations between morphological and behavioral features, supporting multi-parametric toxicity assessment (Adapted from [177,185]).

In practical applications, HTS systems incorporating microfluidic devices or multi-well plate formats can standardize the exposure of *C. elegans* to test compounds. DuPlissis et al. developed an automated pipeline for high-throughput developmental toxicity screening using the vivoChip-24x microfluidic device [185]. Figure 5a,d illustrate the vivoChip-24x setup for *C. elegans* immobilization and imaging, the resulting brightfield and autofluorescence images at different methylmercury concentrations, and the quantitative analysis of body parameters, showcasing the system’s capability for automated body parameter and autofluorescence analysis. This platform immobilizes approximately 1000 *C. elegans* from 24 populations across 40 channels without the use of anesthetics, enabling rapid acquisition of high-resolution 3D images. The image analysis pipeline employs vivoBodySeg, an ML model based on a 2.5D U-Net architecture with attention mechanisms, which achieves 97.80% segmentation accuracy for densely packed worms. Following segmentation, the system classifies worms as complete, partial, or absent, and extracts a comprehensive set of morphometric features, including body length, area, volume, curvature, and fluorescence intensity. Platform sensitivity was demonstrated using a 12-point concentration range of methylmercury (CH_3_Hg), a known neurotoxicant, with EC_10_ values identified as low as 0.46 μM and LOAELs beginning at 1.0 μM. To process these features, the statistical analysis pipeline includes z-score normalization to standardize feature distributions, calculation of coefficients of variation (CV) to assess variability, and application of Tukey fences to identify and exclude outliers. For each well, representing a specific toxin concentration, the system computes the mean and standard error of the mean (SEM) across multiple replicates. Dose–response relationships are modeled using a 4-parameter Hill function to estimate effective concentration values (e.g., EC10). To determine the lowest observable adverse effect level (LOAEL), the pipeline applies Shapiro–Wilk normality tests followed by Welch’s ANOVA and Dunnett T3 post hoc tests to compare treatment groups. This fully automated system processes approximately 36 GB of image data per chip in about 35 min, providing reproducible developmental toxicity indices and supporting rapid, scalable toxicological screening.

### 5.2. AI-Driven Neurotoxicity Assessment Using Multi-Well Plate Screening

In a complementary approach, Currie et al. outlined a high-content imaging pipeline in which synchronized *C. elegans* larvae are sorted into multi-well plates using instruments like the COPAS BIOSORT to ensure standardized compound exposure [177]. High-resolution fluorescent and bright-field imaging is performed using systems such as the Cytation 5 Cell Imaging Multi-Mode Reader to capture both neuronal morphology and behavioral data. AI-driven tools, including DeepImageJ combined with the HPA segmentation model, are then used to extract precise morphological and behavioral features from these images. Techniques such as DL-based segmentation and automated feature extraction allow for quantification of phenotypic changes with high precision. Dimensionality reduction methods, including PCA, are subsequently applied to distill the dataset, retaining essential features that explain the most variance—particularly those capturing key changes in neuronal morphology, synaptic function, and locomotion-. These principal components are then used as robust inputs for ML and DL models, which classify samples into “normal” or “toxic” categories, or generate continuous toxicity scores. Even subtle phenotypic shifts—such as minor reductions in GFP fluorescence intensity indicating altered neuronal activity, or slight changes in locomotion metrics like speed and reversal frequency suggesting early neurotoxic stress—are effectively captured. Moreover, by integrating time-course data from behavioral assays and computing metrics like the area under the curve (AUC), the pipeline accounts for both immediate and cumulative effects of toxicant exposure. This platform was employed to assess the neurodevelopmental toxicity of eleven Per- and polyfluoroalkyl substances (PFAS) compounds commonly detected in drinking water, including 6:2 fluorotelomer sulfonic acid (6:2 FTS), hexafluoropropylene oxide dimer acid (HFPO-DA), perfluorobutanoic acid (PFBA), perfluorobutanesulfonic acid (PFBS), perfluorohexanoic acid (PFHxA), and perfluorooctanesulfonic acid (PFOS). Figure 5e–g illustrates the correlation between toxicity and neurodevelopment, highlighting the effects of PFAS exposure on *C. elegans* behavior, including changes in center point speed, with blue indicating positive and red indicating negative correlations. PFOS and PFBS exhibited significant neurotoxic effects, disrupting dopaminergic neuron activity and synaptic transmission, leading to behavioral deficits such as reduced motility and increased paralysis in aldicarb-induced assays.

## 6. Limitations and Future Directions

As *C. elegans* has long served as a model organism for investigating the physiological effects of environmental toxicants, this multicellular nematode holds strong potential as a living biosensor when integrated with high-throughput microfluidic manipulation systems and AI-driven imaging analytics. To realize such an integrated biosensing platform for real-world environmental monitoring, future research must establish a quantitative and mechanistic correlation between *C. elegans* phenotypic responses and analytical detection metrics, including detection limits, sensitivity, and analyte specificity, to rigorously assess its feasibility for on-site deployment. Because most *C. elegans* phenotypes are characterized through imaging-based approaches, it is essential that imaging outputs be interpreted in terms of actionable environmental indicators, such as the estimated concentrations of target toxicants. As discussed in preceding sections, this connection can be substantially enhanced by AI-based image analysis but must be benchmarked against established environmental monitoring standards to ensure reliability and translational validity. Furthermore, species-specific differences in metabolism and neural processing between the invertebrate *C. elegans* and mammalian systems must be carefully considered to contextualize biosensor readouts in relation to implications in human health. Finally, widespread adoption of such an integrated living biosensor will require system-level standardization, encompassing microfluidic operation, *C. elegans* handling protocols, and image analysis algorithms, alongside a comprehensive evaluation of cost-effectiveness and scalability to support future commercialization.

## Data Availability

This narrative review synthesizes recent advances in *C. elegans* physiology and high-throughput experimental methods that could contribute to *C. elegans*-based environmental sensing. We surveyed literature from PubMed, Web of Science, and Google Scholar, focusing on peer-reviewed articles describing microfluidic platforms, AI-driven phenotyping, and environmental toxicity study applications. Given the interdisciplinary and rapidly evolving nature of this field, we prioritized recent technological integrations over systematic coverage. We acknowledge this approach may not capture all relevant work but provides a focused synthesis of emerging capabilities.

## Abbreviations

The following abbreviations are used in this manuscript:

RTCSM	Real-time continuous soil monitoring
AI	Artificial Intelligence
ERA	Environmental Risk Assessment
COPAS	Complex Object Parametric Analyzer
qPCR	Quantitative Polymerase Chain Reaction
LC-MS/MS	Liquid Chromatography Mass Spectrometry
GC-MS	Gas Chromatography Mass Spectrometry
LC50	Lethal Concentration
LD50	Lethal Dose
6-OHDA	6-hydroxydopamine
MPP^+^	1-methyl-4-phenylpyridinium
GFP	Green Fluorescent Protein
smFISH	Single-molecule fluorescence in situ hybridization
AgNPs	Silver Nanoparticles
MPTP	1-methyl 4-phenyl 1,2,3,6-tetrahydropyridine
DMF	Digital Microfluidics
PDMS	Polydimethylsiloxane
CNN	Convolutional Neural Network
DL	Deep Learning
GAN	Generative Adversarial Network
ML	Machine Learning
CV	Computer Vision
MWT	Multi-Worm Tracker
TMW	Tierpsy Multi-Worm
DWT	Deep-Worm Tracker
YOLO	You Only Look Once
RPN	Region Proposal Network
PCA	Principal Component Analysis
t-SNE	t-Distributed Stochastic Neighbor Embedding
LDA	Linear Discriminant Analysis
ICA	Independent Component Analysis
NMF	Non-negative Matrix Factorization
UMAP	Uniform Manifold Approximation and Projection
PC	Principal Component
SVM	Support Vector Machines
RF	Random Forest
HTS	High-Throughput Phenotypic Screening
LOAEL	Lowest Observable Adverse Effect Level
CV	Coefficients of Variation
SEM	Standard Error of the Mean
AUC	Area Under the Curve
PFAS	Polyfluoroalkyl Substances
PFBA	Perfluorobutanoic Acid
PFBS	Perfluorobutanesulfonic Acid
PFHxA	Perfluorohexanoic Acid
PFOS	Perfluorooctanesulfonic Acid
